# Preparation and Physicochemical Properties of 10-Hydroxycamptothecin (HCPT) Nanoparticles by Supercritical Antisolvent (SAS) Process

**DOI:** 10.3390/ijms12042678

**Published:** 2011-04-20

**Authors:** Xiuhua Zhao, Yuangang Zu, Ru Jiang, Ying Wang, Yong Li, Qingyong Li, Dongmei Zhao, Baishi Zu, Baoyou Zhang, Zhiqiang Sun, Xiaonan Zhang

**Affiliations:** Key Laboratory of Forest Plant Ecology, Northeast Forestry University, Ministry of Education, Harbin 150040, China; E-Mails: xiuhuazhao@nefu.edu.cn (X.Z.); 644677614@qq.com (R.J.); 41611005@qq.com (Y.W.); 724506183@qq.com (Y.L.); qingyong_li@163.com (Q.L.); 156828568@qq.com (D.Z.); zubaishi@163.com (B.Z.); zhangbaoyou@163.com (B.Z.); zhiqiangshun@163.com (Z.S.); 56573995@qq.com (X.Z.)

**Keywords:** 10-hydroxycamptothecin, supercritical antisolvent, nanoparticles, preparation, physicochemical properties

## Abstract

The goal of the present work was to study the feasibility of 10-hydroxycamptothecin (HCPT) nanoparticle preparation using supercritical antisolvent (SAS) precipitation. The influences of various experimental factors on the mean particle size (MPS) of HCPT nanoparticles were investigated. The optimum micronization conditions are determined as follows: HCPT solution concentration 0.5 mg/mL, the flow rate ratio of CO_2_ and HCPT solution 19.55, precipitation temperature 35 °C and precipitation pressure 20 MPa. Under the optimum conditions, HCPT nanoparticles with a MPS of 180 ± 20.3 nm were obtained. Moreover, the HCPT nanoparticles obtained were characterized by Scanning electron microscopy, Dynamic light scattering, Fourier-transform infrared spectroscopy, High performance liquid chromatography-mass spectrometry, X-ray diffraction and Differential scanning calorimetry analyses. The physicochemical characterization results showed that the SAS process had not induced degradation of HCPT. Finally, the dissolution rates of HCPT nanoparticles were investigated and the results proved that there is a significant increase in dissolution rate compared to unprocessed HCPT.

## 1. Introduction

**Figure 1.**
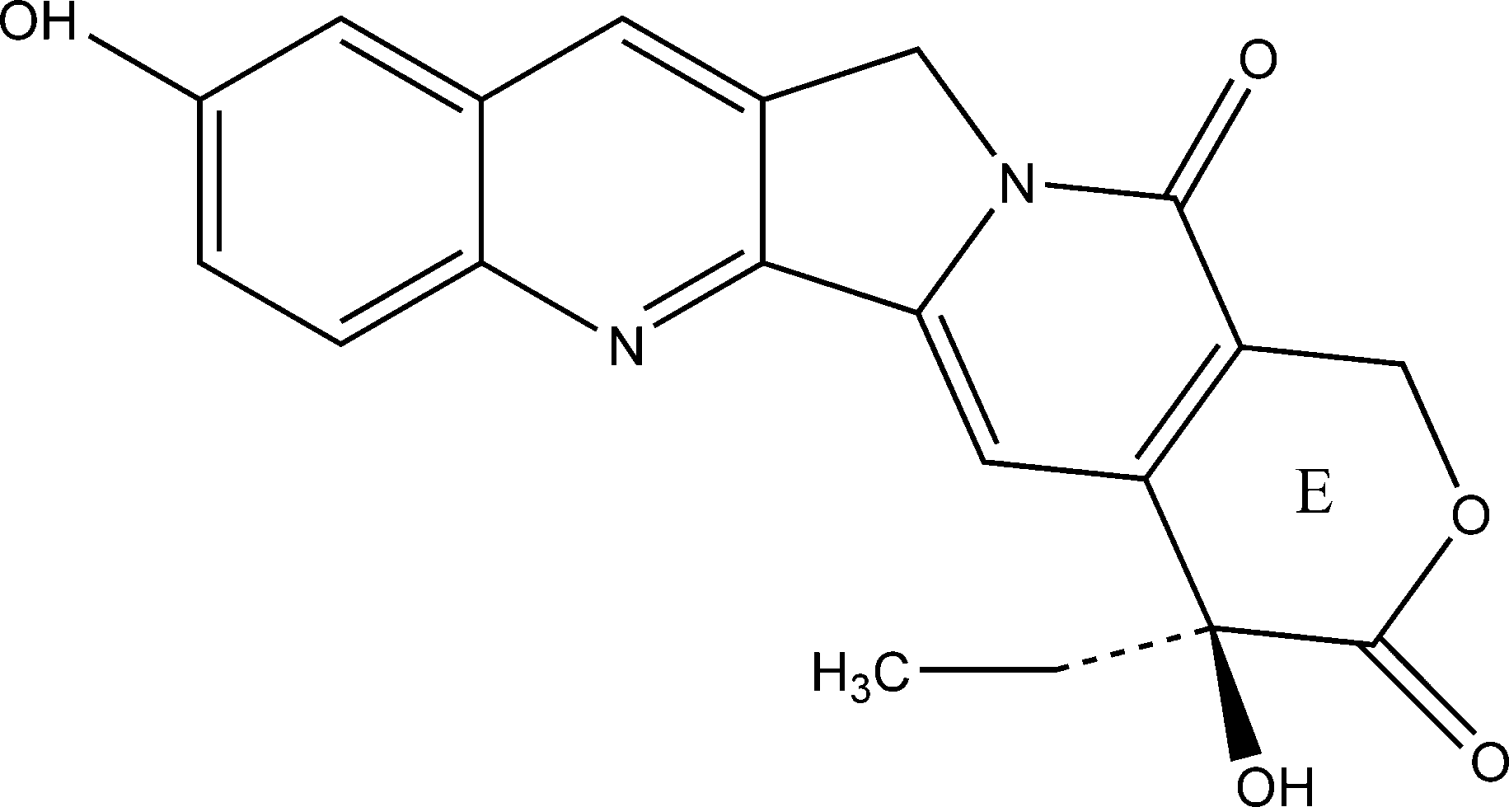
Molecular structure of 10-hydroxycamptothecin (HCPT).

10-Hydroxycamptothecin (HCPT) (Figure 1), one of the natural camptothecin analogues, has been shown to have a broad spectrum of antitumor activity against breast, colon, lung, and ovarian cancers in clinical practice. More importantly, HCPT was shown to be more potent and less toxic than camptothecin(CPT) in experiments on animals and in human clinical evaluations, mainly in China [1,2]. However, due to its poor solubility in water or in physiologically acceptable organic solvents and *in vitro* and *in vivo* instability, clinical practical use of HCPT is limited [3]. The earlier clinical trials using its highly water-soluble sodium salt, HCPT-Na^+^, which was the product of opening the lactone ring of HCPT, showed minimal anticancer activity and several unpredictable side effects such as myelosuppression, hemorrhagic cystitis, diarrhea, nausea, vomiting, and dermatitis. The lactone form is a more effective inhibitor of topoisomerase I compared to the carboxylate form, which has much lower anti-tumor activity [4]. Therefore, it is of great significance in developing the high performance delivery systems for the insoluble lactone form 10-HCPT. Recently, some novel delivery systems of HCPT have been reported, such as nanosuspensions [5], prodrugs [6–9], nanoparticles [10,11], microspheres [12–14], niosomes [3], emulsions [15] and polymeric micelle systems [16,17]. Among these tested approaches, nanosuspensions system, a sub-micro colloidal dispersion system, is considered as one of the most promising methods because of its characteristics in drug loading, release, delivery, stability and various options for administration, such as oral, parenteral, ocular and pulmonary pathways [18]. Production technologies of nanosuspensions currently used are pearl milling and high-pressure homogenization either in water or alternatively in mixtures of water with water miscible liquids or non-aqueous media, such as liquid polyethylene glycols or oils. Supercritical antisolvent (SAS) is a new micronization technology that has been developed in recent years. It is suitable to prepare nanocrystals of drugs or biologically active substances because of their low temperature and inertia. Until now, many bioactive materials have been successfully processed into nanocrystals [19–24].

However, no report has been carried out on the micronization of HCPT using SAS process. Therefore, the aims of this work are to study the feasibility of HCPT nanoparticle preparation by SAS process and to evaluate the SAS process factors which influence the mean particle size (MPS) of HCPT nanoparticles. The effects of concentration of HCPT solution, precipitation temperature, precipitation pressure and drug solution flow rate on the MPS of HCPT nanoparticles were studied by a four-factor, four-level OAD with an OA_16_ (4^5^) matrix. Moreover, characterization of HCPT nanoparticles was analyzed by SEM, DLS, FTIR, LC-MS, XRD and DSC with the purpose of developing a suitable drug delivery system of cancer chemotherapy, nanosuspension of close-ring HCPT.

## 2. Experimental Section

### 2.1. Materials

10-HCPT (98.5% purity) was obtained from Zhejiang Hisun Pharmaceutical Co. Ltd (PR China). High purity CO_2_ (99.99% purity) was purchased from Liming Gas Company of Harbin (PR China). Dimethyl sulfoxide (DMSO, 98.5% purity) was purchased from Sigma Aldrich. All solutions used in HPLC analysis were of HPLC grade and filtered using a 0.22 μm membrane filter (YDHITECH, China) with a filtration system (SHB-III, China).

### 2.2. Apparatus and Supercritical Anti-Solvent (SAS) Process

**Figure 2.**
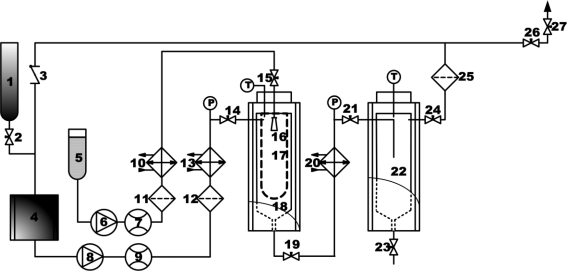
Schematic illustration of the supercritical antisolvent (SAS) experimental apparatus. 1. CO_2_ cylinder; 2. 14, 15, 19, 21, 23, 24, 26 and 27. Valves; 3. Check valve; 4. CO_2_ cooler; 5. Liquid solution supply; 6. Liquid pump; 7 and 9. Flow meter; 8. CO_2_ pump; 10, 13 and 20. Heat exchangers; 11, 12 and 25. Filters; 16. Nozzle; 17. Stainless steel frit vessel of 200 nm; 18. Precipitation chamber; 22. Gas-liquid sepatation chamber.

The schematic of SAS process is shown in Figure 2. The apparatus consists of a precipitation chamber and a gas-liquid separation chamber. The CO_2_ is cooled with a cooler before being compressed by a liquid pump and the pressure is controlled by a back pressure regulator. Afterwards, the CO_2_ is pre-heated in a heat exchanger and enters into the precipitation chamber. Simultaneously, the solution is pumped, heated and fed to the 1000 mL precipitation chamber through a stainless steel nozzle. This nozzle is located in a distinct inlet point from the CO_2_, but also in the top of the precipitation chamber. A stainless steel frit vessel of 200 nm was put into the precipitation chamber to collect the micronized particles and to let the SC–CO_2_/organic solvent mixture pass through. The flow rate of the mixture that leaves the precipitator is controlled by a valve located between the precipitation chamber and the gas-liquid separation chamber. Here the mixture undergoes a decompression (pressure < 5 MPa) to induce the separation of the CO_2_ from the organic solvent. Further information and a schematic representation of the apparatus have been given elsewhere [25].

An SAS experiment begins by delivering supercritical CO_2_ to the precipitation chamber until the desired pressure is reached. CO_2_ steady flow of 8.5 kg/h is established; then pure DMSO is sent through the liquid pump to the precipitation chamber with the aim of obtaining steady state composition conditions during the HCPT precipitation. At this point, the flow of the pure DMSO is stopped and the HCPT/DMSO liquid solution is delivered through the nozzle of 150 μm. Once injected the fixed quantity of HCPT/DMSO solution, the liquid pump is stopped. However, supercritical CO_2_ continues to flow for 30 min to wash the stainless steel frit vessel of 200 nm from the residual content of liquid solubilized in the supercritical antisolvent. If the final purge with pure CO_2_ is not performed, DMSO condenses during the depressurization and redissolves the micronized HCPT.

### 2.3. Optimization of SAS Process

An orthogonal OA_16_ (4)^5^ test design was used to investigate the optimal micronized condition of HCPT. The SAS experiment was carried out with 4 factors and 4 levels, namely: concentration of HCPT solution (0.5, 1.5, 3.0, 5.0 mg/mL), drug solution flow rate (3.3, 6.6, 9.9, 13.2 mL/min), precipitation temperature (35, 46, 57, 68 °C) and precipitation pressure (10, 15, 20, 25 MPa). The range of each factor level was based on the results of preliminary experiments. The mean particle size (MPS) of micronized HCPT (nm) was the dependent variable. The micronized HCPT obtained from the above 16 tests (Table 1) was operated following the method in the Section 3.2.

### 2.4. Physicochemical Properties of HCPT Nanoparticles

#### 2.4.1. Research of the Morphology

The morphology analysis of HCPT particles was carried out using SEM (Quanta 200, FEI). The samples were prepared by direct deposition of the powders onto a carbon tape placed on the surface of an aluminium stub. Before analysis, the samples were coated with gold for 4 min using a sputter coater.

#### 2.4.2. Dynamic Light Scattering (DLS)

Micronized HCPT was suspended in filtered pure water and special cares were taken to eliminate dust and to avoid the aggregation of particles. The water was pre-saturated with HCPT to avoid dissolution of the micronized particles. The suspension was analyzed in DLS (ZetaPALS, Brookhaven Instruments). Every measurement was repeated at least three times.

#### 2.4.3. FTIR Analysis

The HCPT particles were diluted with KBr mixing powder at 1% and separately pressed to obtain self-supporting disks. The FTIR spectrum was obtained by IRAffinity-1 (SHIMADZU, Jepan) and recorded in the wave number range of 4000–500 cm^−1^ at a resolution of 4 cm^−1^.

#### 2.4.4. LC-MS Analysis

The HCPT particles were dissolved in ethanol and LC-MS was obtained by analyst 1.4 of AB API 3000 (USA). The mass spectrometer was operated in positive ion mode.

#### 2.4.5. XRD Analysis

10 mg samples of HCPT particles, forming a weighted dispersion on a glass slide, were evaluated using an X-ray powder diffractometer (Philips, Xpert-Pro, The Netherlands). The samples were irradiated using a Cu target tube at 30 mA and 50 kV. The samples were filled to the same depth inside the sample holder by leveling with a spatula. The scanning rate (5° min.) was constant for all XRD analysis.

#### 2.4.6. DSC Analysis

Thermal analysis was carried out using DSC (TA instruments, model DSC 204) for HCPT particles. All thermal analyses were performed in an inert atmosphere (N_2_). Analysis was performed for 5.0 mg samples at a temperature heating rate of 5 °C/min and a temperature range of 20–265 °C.

#### 2.4.7. Dissolution Studies *in Vitro*

100 mg sample was added to a 500 mL dissolution medium (6.4 g Na_2_HPO_4_, 12H2O; 0.6 g KH_2_PO_4_; 5.85 g NaCl in 1000 mL distilled water; pH 7.4). Bath temperature and paddle speed were set at 37 ± 0.5 °C and 100 rpm. At selected periods of 5, 10, 15, 30, 45, 60 and 120 min, 2 mL aliquots were withdrawn without media replacement, filtered, and analyzed using reversed-phase HPLC (waters) at λ = 266 nm. The data was expressed as a mean value ± S.D (n = 6). The percentage of cumulated dissolution was defined as the sum of the mass of dissolved HCPT at time *t*, divided by the mass of added HCPT (100 mg). The dissolution profiles were plotted as the percentage of cumulated dissolution *versus* incubation time.

Filtered samples were appropriately diluted with methanol and assayed for HCPT concentration by HPLC. Chromatographic analyses were performed on a waters HPLC system consisting of a pump (Model 1525), an auto-sampler (Model 717 plus), UV detector (Waters 2487 Dual λ Absorbance Detector). The C_18_ reverse phase column (Diamonsil, μm, 4.6 mm × 250 mm, Dikma Technologies) was used at room temperature. The mobile phase consisted of 70% acetonitrile delivered at 1.0 mL/min. The injection volume was 10 μL. The signal was monitored at 266 nm.

## 3. Results and Discussion

### 3.1. Optimization Study

The first step in the SAS micronization process is to optimize the operating conditions to obtain a minimum MPS of micronized HCPT. Since various parameters potentially affect the micronization process, the optimization of the experimental conditions is a critical step in developing an SAS method. In fact, the concentration of drug solution, precipitation temperature, precipitation pressure and drug solution flow rate are generally considered the most important factors that affect the MPS of HCPT. The investigated levels of each factor were selected depending on the preliminary experiment results of the single-factor. The optimization of the suitable operating conditions in SAS can be carried out step by step or by using an experimental design. In the present study, all selected factors were examined using an orthogonal OA_16_ (4^5^) test design. The total evaluation index was used to analyse by statistical methods. The analysis results of orthogonal test, performed by statistical software Design Expert 7.0, are presented in Table 1. Table 2 lists the data of the analysis of variance (ANOVA) table of this experiment. The results of experiments presented in Table 2 indicate that the MPS of micronization HCPT varied between 210.5 and 900.7 nm. The maximum MPS was found for trial 16 and the lowest for trial 2. The factors influence the MPS of micronization HCPT were listed in a decreasing order as follows: A > C > B > D according to the *R* value. So, the minimum MPS of micronization HCPT was obtained when the concentration of HCPT solution, precipitation temperature, precipitation pressure and drug solution flow rate were A_1_B_1_C_3_D_2_ (0.5 mg/mL, 35 °C, 20 MPa and 6.6 mL/min), respectively. Through a confirmatory test, smaller micronized HCPT was obtained, with a MPS of 180 ± 20.3 nm.

### 3.2. Effect of Operating Conditions on the MPS of Micronized HCPT

Analysis of variance showed that the concentration of HCPT solution, precipitation temperature and precipitation pressure had a significant effect on the MPS of micronized HCPT (p < 0.05) in the selected ranges (Table 2). However, drug solution flow rate has no significant influence on the MPS.

An orthogonal experimental design allows the separation of the effects of each individual factor investigated. However, it is interesting to analyze the effects of two factors on MPS. A nonlinear regression was applied using Design Expert 7.0 software to plot surface responses. The correlation related the MPS of micronized HCPT to the main variables-concentration of HCPT solution, precipitation temperature and precipitation pressure.

Equation (1) is expressed as follows:

(1) $$\begin{array}{l} Y\;=\;915.8459\;+\;191.1776\times A-8.0994\times B-67.5255\times C\;+\;0.3836\;\times \;A\times B\;-\;4.6189\times A\times C\\ +\;0.3836\times A\times B\;-\;4.6189\times A\times C\;+\;0.2973\times B\times C\;-\;10.4852\times \;A^{2}\;+\;0.0640\;\times \;B^{2}\;+\;1.4603\times C^{2} \end{array}$$

Where *Y* is the MPS of micronized HCPT (nm), *A* is the concentration of HCPT solution (MPa), *B* is the precipitation temperature (°C), and *C* is precipitation pressure (MPa). The correlation coefficient is 0.9591.

**Figure 3.**
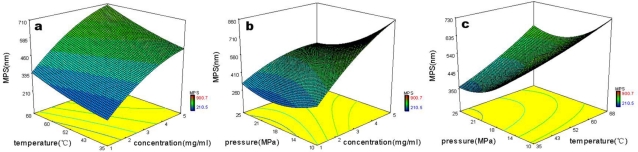
Response surface for MPS of micronized HCPT obtained by nonlinear regression.

Figure 3 shows the three-dimensional response surfaces which were constructed to show the effects of the SAS process variables on MPS of micronized HCPT (*Y*). Figure 3a–c shows the interaction effect of precipitation temperature and concentration of HCPT solution, precipitation pressure and concentration of HCPT solution, and precipitation pressure and precipitation temperature on the MPS, respectively. The MPS of micronized HCPT was found to increase with increasing precipitation temperature and concentration of HCPT solution and decrease with increasing precipitation pressure.

### 3.3. Morphology of Processed HCPT

**Figure 4.**
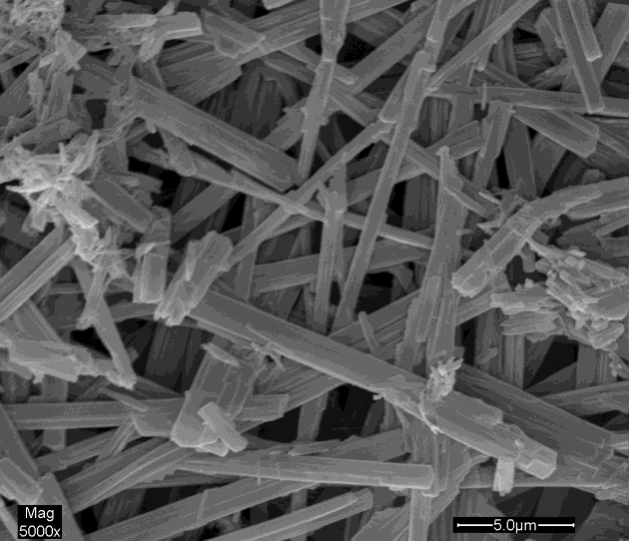
Scanning electron microscope (SEM) image of unprocessed HCPT.

**Figure 5.**
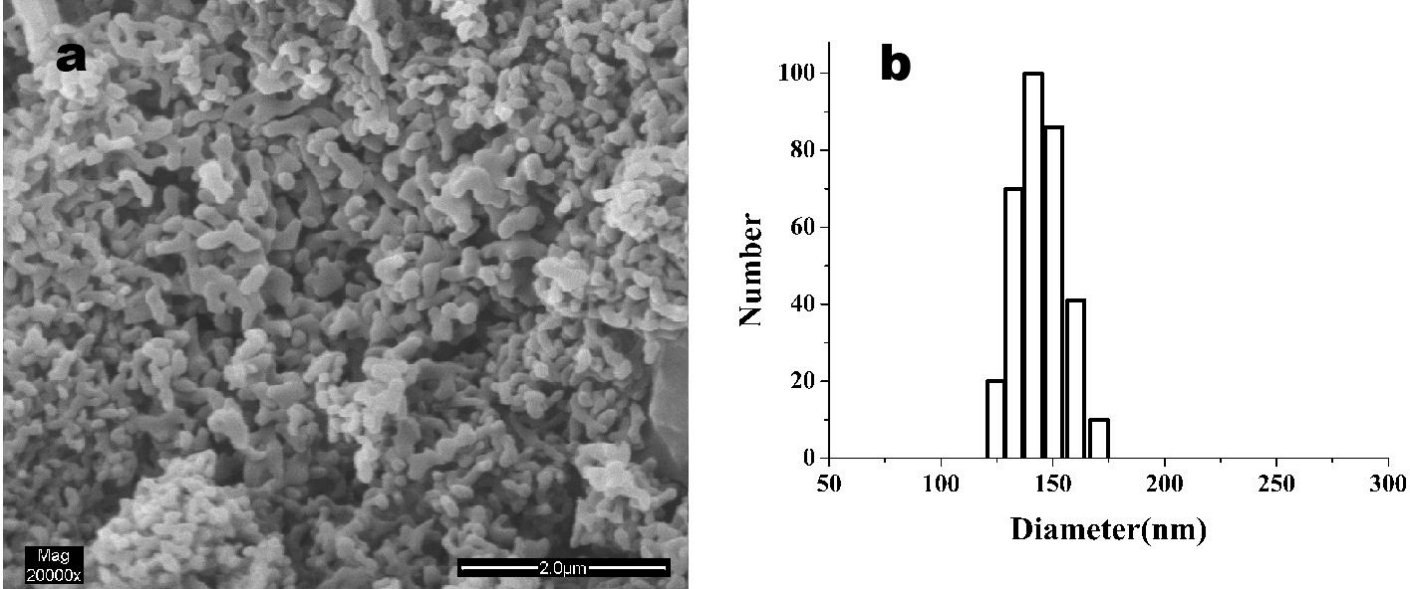
SEM image (**a**) and particle size distribution (PSD) (**b**) of HCPT nanoparticles.

Figure 4 shows the SEM photomicrograph of original HCPT particles. As we can see, commercial HCPT particles are not uniform and basically pillar or bar shaped. The average particle size was found to be about 1 × 1 × 50 μm. Figure 5a shows the SEM images of HCPT nanoparticles precipitated from DMSO under the optimum SAS process condition (0.5 mg/mL of concentration of HCPT solution, 35 °C of precipitation temperature, 20 MPa of precipitation pressure and 6.6 mL/min of HCPT solution flow rate), from which it is obvious that the HCPT nanoparticles are close to ellipsoidal with the MPS of 180 ± 20.3 nm and the particle size distribution (PSD) is shown in Figure 5b.

### 3.4. Physicochemical Properties of HCPT Nanoparticles

#### 3.4.1. FTIR Analysis

**Figure 6.**
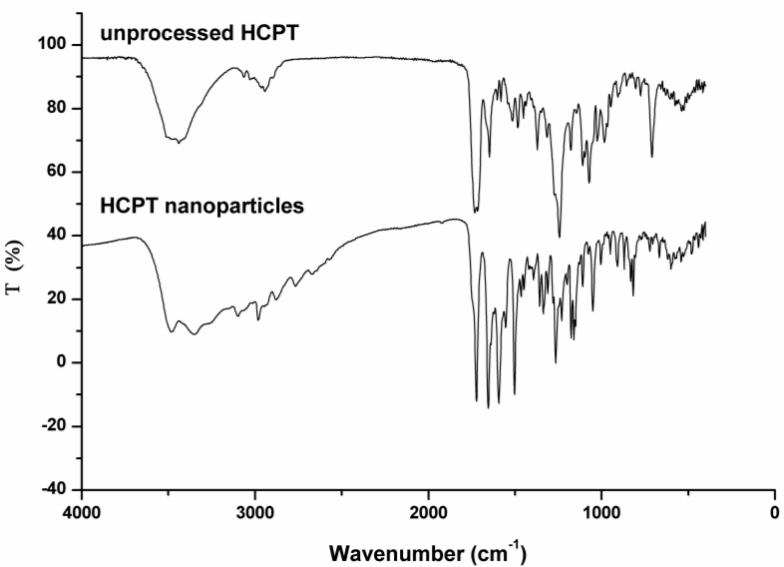
FTIR spectra of unprocessed HCPTand HCPT nanoparticles.

We performed some analysis on original HCPT particles and HCPT nanoparticles to obtain information on the change of chemical structure after SAS processing. It can be seen that FTIR spectra between unprocessed and HCPT nanoparticles do not show any significant differences (Figure 6). Several characteristic IR absorption bands and their assignments are shown as follows: 3494 and 3346 cm^−1^ (the O–H stretching mode), 3100–2750 cm^−1^ (the C–H stretching vibration), 1723 cm^−1^ (C═O stretching vibration of lactone), 1653 cm^−1^ (the C═O stretching vibration of amide), 1593 and 1502 cm^−1^ (the aromatic ring mode), 1266 and 1051 cm–^1^ (the C–O and/or C–N stretching mode).

#### 3.4.2. LC-MS Analysis

**Figure 7.**
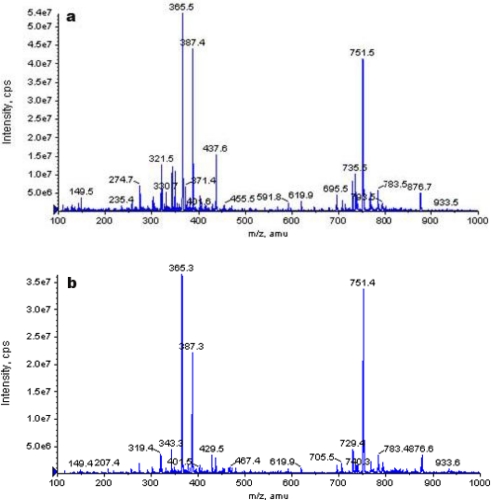
LC-MS spectra of the unprocessed HCPT (**a**) and HCPT nanoparticles (**b**).

The unprocessed and processed HCPT samples were evaluated by using LC-MS to determine the molecular weights, as shown in Figure 7. It can be seen that no modification occurred in molecular weight (364.5). The two forms exhibit the mass spectral peak, at 365.5, coming from the [C_20_H_16_N_2_O_5_ + H]^+^ peak. This explains why there were no varieties about chemistry structure of HCPT before/after SAS process. Therefore, the SAS process has not induced degradation of HCPT.

#### 3.4.3. X-ray Analysis

**Figure 8.**
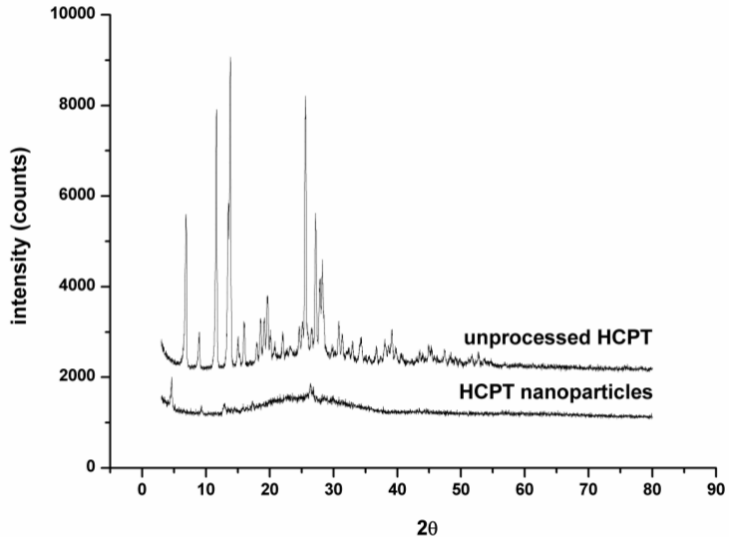
XRD patterns of the unprocessed HCPT and HCPT nanoparticles.

In order to further investigate particles, crystalline XRD analysis was performed. Figure 8 shows the XRD results for unprocessed HCPT particles and HCPT nanoparticles. The presence of several distinct peaks in the XRD of unprocessed HCPT at the diffraction angles of 2θ = 13.821°, 11.659°, 25.640°, 13.521°, 6.900°, 28.260°, 27.919° and 27.219° reveals that the drug is present as a crystalline form. On the other hand, HCPT nanoparticles were characterized by the complete absence of any diffraction peak corresponding to unprocessed HCPT. This fact suggests that HCPT particles after SAS processing are no longer present in crystalline form, but exist in the amorphous state. Less crystalline smaller drug particles are higher in the dissolution rate or bioavailability than crystals and, thus, the therapeutic action is obtained in shorter times.

#### 3.4.4. DSC Analysis

**Figure 9.**
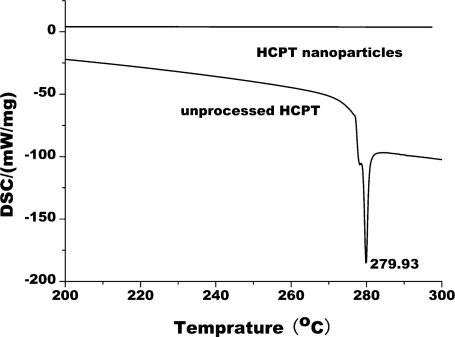
DSC results of the unprocessed HCPT and HCPT nanoparticles.

Figure 9 shows the DSC curves of unprocessed HCPT particles and HCPT nanopaticles. The DSC curves of unprocessed HCPT, a sharp endotherm at 279.93 °C were observed. The sharp endotherm at 279.93 °C might be due to the melting point of HCPT. However, no endotherms were observed in the DSC curves for HCPT nanoparticles. This result indicates that HCPT is no longer present as a crystalline form when processed using SAS, but exists in the amorphous state.

#### 3.4.5. Dissolution Studies *in Vitro*

**Figure 10.**
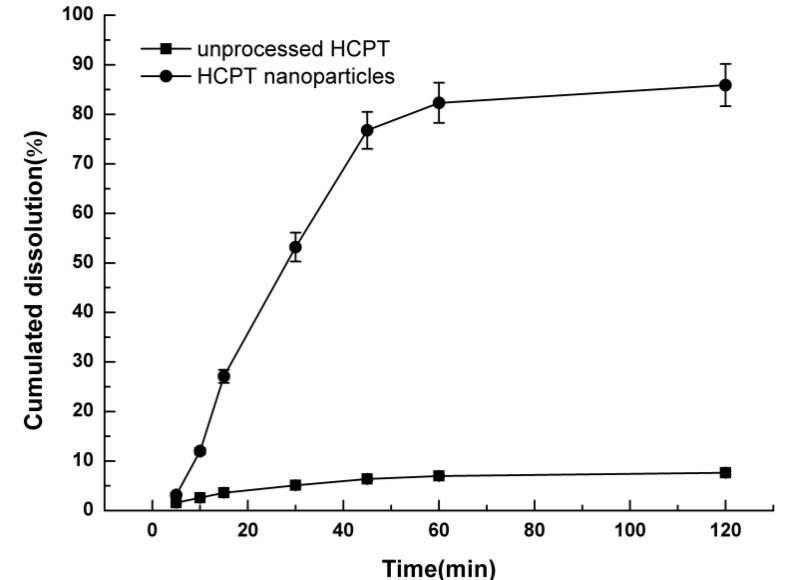
Dissolution profiles of the unprocessed HCPT and HCPT nanoparticles.

Dissolution profiles of HCPT particles are shown in Figure 10. The HCPT nanoparticles showed a more rapid dissolution than the unprocessed HCPT. After 120 min, the dissolution rate of HCPT nanoparticles and unprocessed HCPT were 85.89% and 7.64%, respectively. The HCPT nanoparticles had solubility eleven times higher than the unprocessed HCPT material. The dramatic increase in drug dissolution rate can be explained by the reduction of particle size resulting in an increased specific surface area. The literature has also discussed how an increase in the amorphous content and porosity of the micronized drugs led to an enhanced dissolution rate [26].

## 4. Conclusions

In the present work, a process for micronization of HCPT by supercritical antisolvent (SAS) has been developed. Of the four factors that have an effect on SAS micronization of HCPT from DMSO, three of the factors-concentration of HCPT solution, precipitation temperature and precipitation pressure-have a significant effect on the MPS of micronized HCPT, while the drug solution flow rate showed insignificant effects. The optimum micronization conditions are as follows: 35 °C of precipitation temperature, 20 MPa of precipitation pressure, the flow rate ratio of CO_2_ and HCPT solution 19.55 and 0.5 mg/mL of HCPT solution concentration. Under the optimum conditions, HCPT nanoparticles with an MPS of 180 ± 20.3 nm were obtained. The characterization data of the HCPT nanoparticles using SEM, DLS, FTIR, LC-MS, XRD and DSC showed on one hand, no degradation of HCPT is induced by the SAS process, on the other hand, the obtained HCPT particles have lower crystallinity. Additionally, there is a great increase of dissolution rate in HCPT nanoparticles by the SAS process compared to unprocessed HCPT. In conclusion, the SAS process proposed in the study proved an efficient method for the preparation of HCPT nanoparticles.

## Figures and Tables

**Table 1.** Orthogonal array design matrix L_16_ (4)^5^ and experimental results.

**Trial No.**	**A**	**B**	**C**	**D**	**Error**	**MPS (nm) ± SD (n = 3)**
1	1 (0.5)	1 (35)	1 (10)	1 (3.3)	1	381.2
2	1 (0.5)	2 (46)	2 (15)	2 (6.6)	2	210.5
3	1 (0.5)	3 (57)	3 (20)	3 (9.9)	3	350.0
4	1 (0.5)	4 (68)	4 (25)	4 (13.2)	4	424.8
5	2 (1.5)	1 (35)	2 (15)	3 (9.9)	4	409.4
6	2 (1.5)	2 (46)	1 (10)	4 (13.2)	3	458.3
7	2 (1.5)	3 (57)	4 (25)	1 (3.3)	2	437.9
8	2 (1.5)	4 (68)	3 (20)	2 (6.6)	1	446.0
9	3 (3.0)	1 (35)	3 (20)	4 (13.2)	2	350.6
10	3 (3.0)	2 (46)	4 (25)	3 (9.9)	1	428.2
11	3 (3.0)	3 (57)	1 (10)	2 (6.6)	4	752.8
12	3 (3.0)	4 (68)	2 (15)	1 (3.3)	3	641.1
13	4 (5.0)	1 (35)	4 (25)	2 (6.6)	3	402.9
14	4 (5.0)	2 (46)	3 (20)	1 (3.3)	4	474.5
15	4 (5.0)	3 (57)	2 (15)	4 (13.2)	1	720.6
16	4 (5.0)	4 (68)	1 (10)	3 (9.9)	2	900.7
*K*_1_^a^	341.6	386.0	623.3	483.7	494.0	
*K_2_*	437.9	392.9	495.4	453.1	475.0	
*K_3_*	543.2	565.3	405.3	522.1	463.1	
*K_4_*	624.7	603.2	423.5	488.6	515.4	
*R^b^*	283.1	217.1	218.0	69.0	52.3	
Optimal level	A_1_	B_1_	C_3_	D_2_		

A, Concentration of HCPT solution (mg/mL); B, Precipitation temperature (°C); C, Precipitation pressure (MPa); D, Drug solution flow rate (mL/min)

a *K_i_* = ∑(mean particle size at A_i_)/4, the mean values of MPS for a certain factor at each level with standard deviation.

b *R* = max *K_i_* – min *K_i_*.

**Table 2.** ANONA analysis of four parameters for SAS micronization of 10-hydroxycamptothecin (HCPT).

**Source**	**Sum of squares (SS)**	**Degrees of freedom (df)**	***F*-ratio**	***F_0.05_***	**Type of effect**
(A) Concentration of HCPT solution	182618.6	3	29.038	9.28	Significant
(B) Precipitation temperature	154724	3	24.602	9.28	Significant
(C) Precipitation pressure	117408.4	3	18.669	9.28	Significant
(D) Drug solution flow rate	9585.187	3	1.524	9.28	
Error	6289.02	3			
